# To what extent can traditional medicine contribute a complementary or alternative solution to malaria control programmes?

**DOI:** 10.1186/1475-2875-10-S1-S6

**Published:** 2011-03-15

**Authors:** Bertrand Graz, Andrew Y Kitua, Hamisi M Malebo

**Affiliations:** 1 Geneva University, IMSP/CMU, 1, rue Michel Servet, CH-1211 Geneva 4, Switzerland; 2 Special Programme for Research and Training in Tropical Diseases (WHO/TDR), 20 Avenue Appia, 1211 Geneva 27, Switzerland; 3 National Institute for Medical Research, Ocean Road/Luthuli Street, P.O. Box 9653, Dar es Salaam, Tanzania

## Abstract

Recent studies on traditional medicine (TM) have begun to change perspectives on TM effects and its role in the health of various populations. The safety and effectiveness of some TMs have been studied, paving the way to better collaboration between modern and traditional systems. Traditional medicines still remain a largely untapped health resource: they are not only sources of new leads for drug discoveries, but can also provide lessons and novel approaches that may have direct public-health and economic impact. To optimize such impact, several interventions have been suggested, including recognition of TM's economic and medical worth at academic and health policy levels; establishing working relationships with those prescribing TM; providing evidence for safety and effectiveness of local TM through appropriate studies with malaria patients; spreading results for clinical recommendations and health policy development; implementing and evaluating results of new health policies that officially integrate TM.

## Why focus on traditional medicine as complementary or alternative solutions in malaria control programmes?

The aim of this paper is to discuss past, present and especially future contributions of Traditional medicine (TM) to malaria control programmes. In rural areas in Africa and elsewhere in developing countries, traditional medicines are often trusted, affordable and accessible, as they are made from locally available plants or other elements [1]. A large proportion of the population continues to rely on traditional medicine practitioners and local medicinal plants for primary health care, as a choice or when there is no access to other medicine [1-3].

This reflexion article is an attempt to show trends in TM research and practice in order to answer the following question: should TM be officially considered and recommended as complementary or alternative solutions in malaria control programmes? And, if so, under what conditions?

The usual reason for interest in TM stems from the recognition that two of the major anti-malarial drugs, quinine and artemisinin trace their origin to traditional medicine [4-7]. The efficacy of artemisinin derivatives has prompted the use of the original plant, *Artemisia annua*, as a herbal tea against malaria [8]. The development of traditional herbal medicines for malaria, bioscreening and modern drug development are not mutually exclusive.

Patients use TM for many reasons: They may be in a remote area where modern medicine is not available when they need it. They may belong to communities whose habits and treatment-seeking behaviour resorts to TM as the first choice. They may prefer TM, believing, for example, that they produce fewer side effects or cures them more effectively. They may have experienced a failure with a modern treatment and want to try TM. They may want to avoid modern health facilities because they perceive them as expensive, unfriendly, dangerous or ridden with corruption. Patients may also avoid modern drugs sold on the market because they are aware of the fact that many of them are counterfeit or ‘fake’ drugs .

Some civilizations, e.g. in China and in India, still practice ancient traditional medicines. Ancient use of plant medicine has evolved into 'new' practices such as naturopathy, homeopathy, aromatherapy and modern herbal medicine [10], although many such practices have not been formally validated.

Ethnomedical studies provide information on the use, preferences and ecological viability of plants. For example, 16 plants against malaria were found in the communities living around the Sango Bay Forest Reserve in southern Uganda [11]. *Hallea rubrostipulata*, *Warburgia ugandensis* and *Syzygium guineense* were the most important forest tree species used to treat malaria in the community. Such studies cannot, however, tell us anything about the effectiveness of these plants. For that, clinical studies are necessary.

The term “validated”, in clinical practice and health policy, means that there is enough evidence in clinical studies to ensure safety and effectiveness (i.e. “evidence-based medicine”). Is there anything like “evidence-based TM”? Many physicians are unaware that clinical research has already been conducted and published on TM. Although the vast majority of published material on TM is laboratory research (e.g. phytochemistry or animal studies), there is also today some clinical evidence of effectiveness in this domain [12].

Once a TM has been validated, it could be tested – and in case of good results officially recommended -- as an alternative to modern treatments when the latter are lacking, e.g. when supply is interrupted for logistic reasons. Since very few clinical trials on safety and efficacy of traditional anti-malarials have been conducted [12,13] , the therapeutic potential of TM may well have been underestimated. So far safety and effectiveness of some TMs have been observed, paving the way to better collaboration between modern and traditional medical systems. In addition, health system research has uncovered interesting facts about TM, e.g. that TM practitioners are not responsible for late seeking modern treatment, at least in some settings [14].

TMs appear today a largely untapped health and economical resource, with potential importance at both the individual and population levels[15].

## Herbal compounds provided important leads for modern anti-malarials: quinine and artemisinin

Among the earliest written records of anti-malarial treatments are a series of Babylonian clay tablets from about 2600 B.C [10]. Amongst the approximately 1,000 plant-derived substances cited for various ailments were oils of *Cedrus* species (cedar) and *Cypressus sempevirens* (cypress), *Glycyrrhizza glabra* (licorice), *Camphora* species (myrrh), and *Papaver somniferum* (poppy). All these plants are still used today for the treatment of ailments ranging from coughs and colds to parasitic infections and inflammations [16].

Malaria was a scourge in much of Europe until about three centuries ago. After the Spanish and Portuguese colonization of South America, the use of the bark of the *Cinchona* tree to treat malaria was introduced in Europe, leading to the discovery of quinine, at the beginning of the 19^th^ century [17]; it remains the oldest anti-plasmodial drug still in clinical use. Quinine has perhaps saved more lives than any other drug known in history [4,5]. It has also provided a lead template to chemists who successfully synthesized aminoquinoline-based anti-plasmodial analogs, such as chloroquine, amodiaquine, primaquine and mefloquine, all of which considerably improved the treatment of malaria.

Pre-modern China is also a source of information about the early medicinal uses of plants . The Chinese traditional herbal remedy Qinghao (an *Artemisia*) has long been used in China for the treatment of intermittent fevers. It was first noted in a document found in a tomb dating from 168 B.C., while the first recipe against intermittent fevers in their acute phase was made by Ge Hong in the fourth century AD. In 1596, Li Shizen introduced Ge Hong's recipe into the materia medica literature [18-22].

The emergence of chloroquine-resistant *P. falciparum* malaria in southeast Asia in the 1960s caught the attention of the Chinese government [23]. A National Steering Committee on Antimalaria Research was established in 1967 and an anti-malarial drug discovery programme – encouraged by a war-time request from North Vietnam – was set up in 1969. As part of the programme, Chinese scientists examined ancient medical texts and were drawn to qinghao by Ge Hong’s comments [24]. They confirmed the anti-malarial potency of *Artemisia annua* in 1971, developed an effective extraction process, isolated and identified artemisinin as the active ingredient in 1972 [20,25,22]. Artemisinin, which is effective in treating chloroquine-resistant cases, is quite different from the old generation of anti-malarial drugs because of its peroxide group [19,26]. Attempts to derivatise artemisinin were done even before its chemical structure was fully elucidated. Chemists successfully semi-synthesized anti-plasmodial analogs, such as dihydroartemisinin, artemether, arteether, artesunate and artelinic acid. The derivatives of artemisinin are widely recommended today [27].

## Traditional practitioners recommend referral for severe cases

A study in Tanzania showed that increasing the collaboration between traditional healers and modern health care providers has improved the management of severe malaria [14]. Traditional healers form a natural extension of the formal health service and there is great potential for improving both their practice and access to modern medicines through their training and collaboration.

That is why it could be advisable to start with a collaborative research project on the actual effectiveness of some local TM practices, and only then discuss limitations of local care and the necessity for referral of a few cases. TM practitioners in both Tanzania and Ghana were very cooperative in referring severe cases of malaria during the recently completed rectal artesunate trial, when they were made aware that such cases are treatable and recover well if referred early [28]. Indeed experience shows that TM practitioners are, at this point, ready to discuss referral and happy to improve their competence in this matter.

In most cases, patients actually perform their own self-referrals, usually in quite an appropriate manner. They welcome support in decision making from traditional practitioners. In a study in Mauritania, it appeared that TM practitioners performed adequate referrals without any previous training and were able to predict patient progress as accurately as modern physicians, an observation that was attributed to their long clinical experience [29]. They were able to detect those patients having a high chance of cure with their treatment, and those with a low chance. For the latter, they asked their modern colleagues whether they had something to offer. Joint meetings were organized where modern and TM practitioners presented their difficult patients and asked their counterpart whether they had something to offer. Appropriate referral was facilitated by such trusting relationships.

## Traditional medicine for malaria control programmes in the future

Today, TM can still inspire new therapeutic ideas. Some recipes are being locally validated and will be tested as part of malaria control programmes [30]. In a longer term perspective, the TM approach described here can also be used as a “reverse process” of the classical drug discovery process; it takes advantage of experience gathered during centuries in areas where local remedies are used. Where numerous traditional recipes exist for the same ailment, we can discover the one with the best outcome, which may represent the most promising treatment concept. Approaching TM with the objective of having a net positive impact on malaria control activities is, however, paved with dangers of doing more harm than good.

A prerequisite is to have a multidisciplinary team with excellent technical and human competences, led by a health professional with experience and rigor in clinical research, excellent communication skills and demonstrating a relativist perspective of various medical knowledge, allowing for a positive and critical view on all medicines.

A project of TM for malaria control programme could be organized along the following lines, this suggestion being based on field experience:

## Step 1:	 Recognize TM as a (sometimes) valuable health resource

To start with, members of a team working on TM for malaria control should be aware of the literature and realize that TM may contribute to malaria control programmes in several ways:

- TM practitioners can help in referring severe cases as well as ensuring that pregnant women receive their intermittent treatment adequately;

- TM may provide validated first-aid treatment for rapid care in remote areas;

- the use of TM as first-line treatment for semi-immune population could delay resistance of Plasmodium against the most potent modern anti-malarials;

- new leads for future malaria drugs can be found in local TM.

A common worry with TM is that doses vary. In addition to variations in the mode of preparation, the biochemical content of the plant used is not stable across time and space. The answers to this problem are manifold: for a safe and effective treatment without a precise and stable dose of active constituents, the therapeutic range of the selected preparation must be large enough to allow for wide dose variations, so that the TM retains enough clinical effect without excessive toxicity. It may also be possible to find the active constituents for quality control, a research programme in itself.

- Traditional healers themselves, if reliable and credible, can be a valuable human resource. The ratio of population per healer is much smaller than the ratio of population per nurse or doctor in many areas. Yet the healers are often overlooked in statistics on human resources. Cuba is an excellent example of integrated traditional and modern medicine in the health care system with mutual benefits [31].

- The issue of "counterfeit" drugs can be a reason why TM is preferred, but counterfeit TM does exist as well. Therefore, a form of quality assurance must be put in place (e.g. ensuring that the plant is harvested properly and not confused with others) and work synergistically with existing quality assurance mechanisms for modern medicine.

- Traditional medicines do convey a potential for economic development and poverty reduction through the involvement of other sectors like agriculture, marine resources and forestry. Developing large-scale commercial cultivation of medicinal plants and algae or fungi may provide income to communities and preserve traditional medicinal knowledge and biodiversity.

## Step 2:	Foster working relationship with those using TM

- Health practitioners and physicians or nurses use different tools for a common aim: health. It is, therefore, no surprise that field experience showed that practitioners of different cultures can find a common language when consulting together [29]. As awareness of the similarities between them grows, they can create a good working relationship, even when their diagnostic and therapeutic tools differ widely.

- Both TM and modern practitioners can improve their knowledge. For example traditional practitioners could receive standard training in anatomy, physiology and clinical medicine while modern health practitioners receive training to appreciate the value and potential of TM. This would improve their understanding of each other and their ability to collaborate adequately.

- TM practitioners are usually included in the following research question: “Among all the different local treatments for a given ailment, which is the most effective?” When TM is practiced to some degree in every family, which is usual, the entire community is concerned with a research project aiming at answering a question of practical interest locally.

## Step 3:	 Search for safe and effective local TM

- In the case of *Artemisia annua*, the question has been asked: why not use the plant itself? The hypothesis was that it could prevent drug resistance because it is a “combination therapy” [32]. Phytochemical studies showed that the content of artemisinin may seem quite low, even in selected species, but this depends on the extraction method and several active constituents could be working in synergy. Results of clinical studies are promising, however with relatively high recrudescence rates [33]. Some NGOs have started to encourage local cultivation of *A. annua*[8], that grows easily in humid climates, but not well in dry areas. Another anti-malarial plant, validated in clinical studies, is *Argemone mexicana*[30]; since it grows precisely in the pan-tropical dry belt (Sahel-type climates), the two plants may become an example of complementary solutions, geographically speaking, if further studies confirm their potential in malaria control.

- After preliminary studies for selection of the best candidate, there is a methodological dilemma: designing a randomized controlled trial (RCT) in standard experimental conditions or studying the “real world” situation (or at least ‘as real as possible’). A standard RCT provides sound information on treatment effects, but is usually designed with inclusion criteria that are far removed from the actual indications for malaria treatment in real clinical situations. As a result, external validity may be low. If the goal is to gain a reasonable estimate of the effects of a TM recipe if it is to be recommended for use in home-based or dispensary-based management of malaria, then it will be more appropriate to choose a study design that compares two strategies for the management of presumed uncomplicated malaria on site: TM as first-line with modern treatment (e.g. ACT) as second-line, versus modern treatment as first-line. The main endpoint could be incidence of severe malaria, as a predictor of malaria mortality - or the latter directly if sample size makes it possible.

- TM testing with patients is made easier than with modern drugs, because a good documentation of traditional use alleviates the constitution of the file, provided that the study is conducted with the TM recipe prepared and applied according to the local traditional use [35]. This is also specified in the WHO document “Guidelines for Clinical Study of Traditional Medicines in the WHO African Region” [36]. Legal requirements, of course, vary from country to country.

## Step 4: Spread results for clinical recommendations

Results of clinical studies of TM can be important for different groups:

- For the general public, evidence-based information will help patients and family make better treatment choices. For example, they may know better if a certain TM is a good alternative to modern treatment in some occasions or if it should be avoided.

- For health administrations, the challenge is to have a constructive collaboration between traditional and modern health systems. Activities can be:

◦ Quality control of TM recipes and preparations sold in the country [37]

◦ Assessment of TM practitioners, certification

◦ Support for clinical research and official validation of safe and effective TM

◦ Support for drug discovery based on local recipes, through phytochemistry, in vitro and in vivo studies, patenting and licensing for drug development

◦ Production of TM of officially controlled quality

◦ Pharmacovigilance on TM products

- For health professionals and academics, in an era of pluralistic medicine, conventional (academic) medical centres need to offer basic knowledge on TM and other “alternative and complementary medicines” (CAM) for future health professionals, in order to allow then to inform patients properly on potential usages, dangers and interactions of TM/CAM. Acting at this level may well be the intervention with the longest-lasting effect in terms of spreading more rational views on TM. Until recently, most medical schools tended to teach only one type of medicine. For example in India, there were western, Ayurvedic, or Unani medical schools. In many developing countries, there were only western-style medical schools. Today, there is a growing tendency to integrate teaching on TM into medical courses, focusing on clinical research results and the way to rapidly find such information in available databases. Teaching and research on TM/CAM in medical school can improve the relationship between academic and other practitioners. Universities also have a growing number of doctoral students who choose to conduct their master or doctoral thesis research on TM. These doctoral students play a very important role in the production of scientific knowledge on TM.

## Step 5:	 Make TM part of the public health system

**Figure 1:**
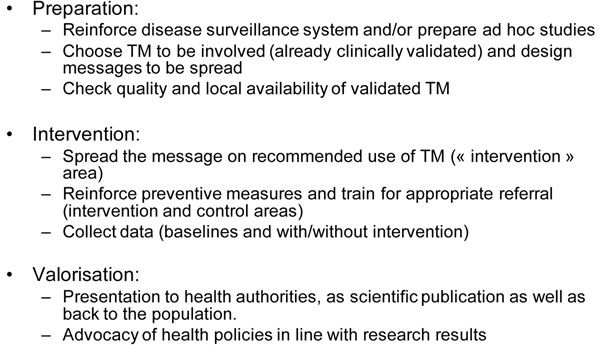
Suggested design of a pilot public health malaria control programme involving traditional medicine.

If research results on TM are taken seriously, they should lead to health policy changes, at least as pilot interventions in the field. Such pilot interventions may show whether a careful integration of TM in health systems where it was not taken into account will have an impact on health indicators (Figure 1). In the case of malaria, a national health policy could introduce, at the district level, recommendations for using a validated TM treatment as first-line treatment for uncomplicated malaria.

Could wide use of validated TM be a way to delay resistance to modern anti-malarials? A classic way of delaying drug resistance is to use an alternative when possible. While resistance to amodiaquine and sulphadoxine-pyrimethamine is already widespread [38,39], there is also some evidence of resistance to lumefantrine [40]. Resistance will continue to spread if last-generation anti-malarials are universally used. Some patients, however, may not absolutely need them, because they are semi-immune. In a longitudinal study in Senegal, clinical outcomes were not worse when old treatments with some parasite resistance were used. Even though resistance to chloroquine (CQ) rapidly increased from 1992 to 2001, no change in CQ prescription was observed until the early 2000s, and this was related to the absence of an obvious decrease in clinical CQ effectiveness [41].

## Step 6: TM potential for lead chemical compounds and drug development

Although this is a logical component of this section, it will not be covered here, because it has been discussed extensively in other articles of this supplement.

One point, however, must be stressed: determination of active compounds has a first, immediate utility in the process described above, it provides the basis for quality control methods. For example, Eritrean public health professionals sent samples of *Argemone mexicana* collected near Asmara in order to know if their plant had an active compounds profile comparable to Malian batches, and thus could be tried as a locally-produced anti-malarial.

Finally the involvement of other sectors in determining the economical value of TM and fostering commercial production of TM products will not only create wealth and reduce poverty, but will also protect biodiversity for future generations.

## Conclusion

The WHO Regional Committee for Africa strategy for promoting TM includes the development of local production and conservation of medicinal plants, legislation of TM practice and its integration into conventional health services.

Despite extensive investments in malaria control, research and elimination programmes, the disease has remained a major public health problem in sub-Saharan Africa and in many other places. Implementing rational use of traditional medicine against malaria through community-based participatory approaches is feasible and may well help curb the toll of the disease in endemic areas. There is an urgent need for major research investments in TM clinical and public health to further develop appropriate solutions for mass application.

## Competing interests

The authors declare that they have no competing interests.

## Authors' contributions

Bertrand Graz wrote the main parts of the first draft, Hamisi Malebo other parts, then the whole was extensively worked on by all three authors.
